# Editorial: Recent advances in the design of novel polymer nanoagents for cancer theranostics

**DOI:** 10.3389/fchem.2023.1216173

**Published:** 2023-05-16

**Authors:** Hanlin Ou, Fan Huang, Bo Shen, Zhenbo Gao, Feihe Ma, Devleena Samanta

**Affiliations:** ^1^ State Key Laboratory of Bio-Fibers and Eco-Textiles, Collaborative Innovation Center of Marine Biobased Fiber and Ecological Textile Technology, College of Materials Science and Engineering, Qingdao University, Qingdao, China; ^2^ Key Laboratory of Radiopharmacokinetics for Innovative Drugs, Chinese Academy of Medical Sciences, Institute of Radiation Medicine, Chinese Academy of Medical Sciences and Peking Union Medical College, Tianjin, China; ^3^ Department of Chemistry, Northwestern University, Evanston, IL, United States; ^4^ Department of Chemistry, College of Sciences, Nanjing Agricultural University, Nanjing, Jiangsu, China; ^5^ State Key Laboratory of Separation Membranes and Membrane Processes and School of Materials Science and Engineering, Tiangong University, Tianjin, China; ^6^ Department of Chemistry, The University of Texas at Austin, Austin, TX , United States

**Keywords:** polymer nanoagent, cancer theranostics, chemiluminescent probe, peptide nanomedicine, extracellular vesicle, smart drug delivery system

The significance of early and precise cancer theranostics in reducing patient mortality has been well established (Tringale et al., 2018; Crosby et al., 2022). In recent years, nanoagents have emerged as a promising tool for cancer theranostics, owing to their passive and active targeting effects on cancerous tissues (Torchilin, 2011). Among the various types of nanoagents, polymer nanoagents have gained increasing attention due to their excellent biocompatibility, ease of processability, and flexible surface functionalization (Ou et al., 2019). In this Research Topic, 9 published articles focus on the design of novel polymer nanoagents for cancer theranostics, providing valuable insights into the development of effective and precise cancer therapies using polymer nanoagents.

One review in this Research Topic, Zhang et al. summarized the recent progress of chemiluminescent polymer nanoagents based on various chromophore substrates, including luminol, peroxyoxalates, 1,2-dioxetanes and their derivatives, for cancer detection. The authors emphasized the design strategies, mechanisms, and diagnostic applications of representative chemiluminescent polymer nanoagents. Huang et al. reviewed the general advances of polymer nanoagents and presented their applications in cancer diagnosis, treatment, and theranostics, respectively. The authors also proposed the challenges and prospects of polymer nanoagents in the cancer theranostics field. As the human tongue can extend out of the mouth and is approximately 1 cm thick, the utilization of polymer nanoagents in oral cancer theranostics is less restricted by tissue depth and is easier to apply in clinical practice. Zhang et al. focused on the application of polymer nanoagents in the diagnosis and treatment of oral cancer. As one of the most frequently used clinically approved biodegradable polymers, poly (lactic-co-glycolic acid) (PLGA)-based nanoagents have been extensively applied in the field of cancer theranostics, including emerging cancer immunotherapy (Koerner et al., 2021). Gao et al. reviewed the advances of PLGA-based nanoagents in cancer immunotherapy, focusing on cancer vaccines and tumor microenvironment modulation.

In recent years, peptide-based nanoagents have gained extensive attention from researchers due to their high specificity and low systemic toxicity (Habibi et al., 2016). Focusing on peptide-based nanoagents, Zeng et al. summarized the applications of peptide-based nanoagents in the diagnosis and treatment of bladder cancer. As a class of lipid membrane-bound vesicles released by various cells, extracellular vesicles (EVs) with high physiochemical stability and biocompatibility have emerged as a new kind of drug delivery system for cancer theranostics (Bose et al., 2018). Nan et al. reviewed the direct modification of EVs and their application for cancer theranostics. The authors highlighted the prevailing approaches for direct modification of EVs, including cargo loading strategy for EVs modification and modification approaches of the EVs membrane. For conventional chemotherapy drugs, their poor tumor targeting effect is a key factor limiting their effectiveness in cancer treatment (Zeng et al., 2021). To address this issue, Rana et al. outlined the design of Smart Drug Delivery Systems (SDDSs) and their application in the cancer theranostics field. SDDSs that respond to redox, enzyme, light, ultrasound, and magnetic stimuli are highlighted. The authors proposed that SDDSs could become the future of Translational Medicine.

In an original research article in this Research Topic, Sun et al. prepared ROS-responsive Amplex^®^ Red (ADHP) nanoprobes and studied their application in image-guided tumor resection, which has not been explored much. ADHP probes can rapidly oxidize in response to ROS in the tumor microenvironment to form resorufin, which significantly reduces the background fluorescence signal compared to the single resorufin probe. With the fluorescence guidance of ADHP nanoprobes, the authors successfully carried out image-guided surgery of 4T1 abdominal tumors. In addition, in response to the severe toxicity and gastrointestinal side effects of colchicine that limit its application in cancer treatment, Li et al. designed and synthesized a novel colchicine-magnolol hybrid (CMH) by splicing colchicine and magnolol, a multifunctional polyphenol showing favorable gastrointestinal protection. The antitumor results showed that CMH inhibited the growth of Lewis lung carcinoma (LLC) cells 100 times more potently than cisplatin, while the cytotoxicity of CMH was 10-fold lower than that of colchicine in normal human lung cells. Western blot results revealed that CMH dose-dependently suppressed the protein expression of phosphorylated ERK.

In conclusion, the rational design of polymer nanoagents, including inner probes/small molecule drugs and the outer polymer matrix (synthetic polymers, peptides, exosomes), plays a significant role in the cancer theranostics effect of polymer nanoagents. To further improve the effectiveness of polymer nanoagents in cancer theranostics, follow-up research could focus on developing smart probes/small molecule drugs with higher sensitivity and tumor responsiveness, and polymeric matrices with enhanced biodegradability and tumor targeting effects. In addition, the biosafety and ease of preparation of polymer nanoagents also need to be considered to make them clinically applicable, which needs to be balanced with the intelligence of polymer nanoagents. We expect that this Research Topic will inspire the design and synthesis of more advanced polymer nanoagents and promote their widespread application in cancer diagnosis and treatment.
